# Role of the Inflammasome-Caspase1/11-IL-1/18 Axis in Cigarette Smoke Driven Airway Inflammation: An Insight into the Pathogenesis of COPD

**DOI:** 10.1371/journal.pone.0112829

**Published:** 2014-11-18

**Authors:** Suffwan Eltom, Maria G. Belvisi, Christopher S. Stevenson, Sarah A. Maher, Eric Dubuis, Kate A. Fitzgerald, Mark A. Birrell

**Affiliations:** 1 Respiratory Pharmacology, National Heart and Lung Institute, Faculty of Medicine, Imperial College London, London, United Kingdom; 2 Hoffmann-La Roche Inc., pRED, Pharma Research & Early Development, DTA Inflammation, Nutley, New Jersey, United States of America; 3 University of Massachusetts, Division of Infectious Diseases & Immunology, Worcester, Massachusetts, United States of America; Fundação Oswaldo Cruz, Brazil

## Abstract

**Background:**

Chronic Obstructive Pulmonary Disease (COPD) is an inflammatory airway disease often associated with cigarette smoke (CS) exposure. The disease is increasing in global prevalence and there is no effective therapy. A major step forward would be to understand the disease pathogenesis. The ATP-P2X_7_ pathway plays a dominant role in murine models of CS induced airway inflammation, and markers of activation of this axis are upregulated in patients with COPD. This strongly suggests that the axis could be important in the pathogenesis of COPD. The aim of this study was to perform a detailed characterisation of the signalling pathway components involved in the CS-driven, P2X_7_ dependent airway inflammation.

**Methods:**

We used a murine model system, bioassays and a range of genetically modified mice to better understand this complex signalling pathway.

**Results:**

The inflammasome-associated proteins NALP3 and ASC, but not IPAF and AIM2, are required for CS-induced IL-1β/IL-18 release, but not IL-1α. This was associated with a partial decrease in lung tissue caspase 1 activity and BALF neutrophilia. Mice missing caspase 1/11 or caspase 11 had markedly attenuated levels of all three cytokines and neutrophilia. Finally the mechanism by which these inflammatory proteins are involved in the CS-induced neutrophilia appeared to be via the induction of proteins involved in neutrophil transmigration e.g. E-Selectin.

**Conclusion:**

This data indicates a key role for the P2X_7_-NALP3/ASC-caspase1/11-IL-1β/IL-18 axis in CS induced airway inflammation, highlighting this pathway as a possible therapeutic target for the treatment of COPD.

## Introduction

Chronic Obstructive Pulmonary Disease (COPD) is an airway inflammatory disease which is increasing in prevalence and predicted to be the third leading cause of mortality by 2020 [1]. It has a significant impact on quality of life and is a major socio-economic burden. Despite this there is currently no therapy available to stop the decline in lung function and disease progression and, therefore, an urgent need to develop effective therpaies [2], [3]. Current dogma suggest that exposure to inhaled pollutants, such as cigarette smoke (CS), drives the chronic inflammation associated with the disease and the subsequent pathophysiological changes in the airway and associated symptoms [4]. Thus a medication that attenuates the airway inflammation should slow disease pathogenesis and reduce symptoms. Therefore, understanding the mechanism by which CS causes the airway inflammation could highlight possible targets for drug development.

Recently there has been growing evidence to implicate the ATP-P2X_7_-inflammasome-caspase 1-IL-1/18 axis in murine models of smoke induced airway inflammation, healthy smokers and in patients suffering from COPD. ATP levels have been reported to be increased in the lungs of smoke driven models and COPD patients [5]–[7]. Activation of the P2X_7_ receptor has been shown to be central to smoke induced airway inflammation [8], [9]. Caspase 1 activity has been reported to be increased in the lungs of the CS-driven models and of COPD patients [8], [10]; IL-1/18 levels are increased in model systems and smokers/COPD patients [10]–[22]. Further, genetic analysis of susceptibility to COPD has been associated with altered IL-1/18 genes [23]–[26]. The aim of this study was to perform a detailed assessment of the signalling post P2X_7_ receptor activation in a CS-driven model. Utilising bioassays and genetically altered mice (KO) we determined the role of the various inflammasome proteins reported to be associated with the P2X_7_ receptor and IL-1/IL-18 maturation i.e. NALP3 (or NLRP3, PYPAF1, CIAS1), AIM2, IPAF (or NLRC4, Pycard, TMS1) and ASC [27]–[29]. Furthermore, the role of caspase 1 and its reported activator caspase 11 [30]–[34] in the release of mature cytokines and the role of each of the three inflammatory IL-1 cytokines: IL-1α, IL-1β and IL-18 was also investigated We also performed parallel experiments in an endotoxin (LPS) model which also has a predominately neutrophilic phenotype to determine if these signaling proteins were required for airway neutrophilia *per se* or specifically after CS challenge.

## Methods

### Mice

All *in vivo* protocols were approved by Imperial College London ethical review process committee and we strictly adhered to the Animals (Scientific Procedures) Act 1986 UK Home Office guidelines. Experiments were performed under a Home office project licence (PPL 70/7212). Male C57bl/6 mice (18–24 g) were originally obtained from Harlan UK Limited (Bicester, UK) and bred in-house; food and water supplied *ad libitum*. KO mice were back crossed at least 8 times and bred alongside the wild type mice: ASC -/-, NALP3 -/-, IPAF -/- IL-1β -/-, IL-1α-/-, IL-18 -/-, IL-18R -/-, caspase 1/11 -/- and caspase 11 -/-. The KO mice were donated from various laboratories: caspase 1/11 -/- from the Swiss Immunological Mouse Repository (SwImMR); IL-1β -/- and IL-1α -/- from Professor Yoichiro Iwakura from the University of Tokyo, IL-18 -/- were from Jackson labs, USA; ASC -/-, NALP3 -/-, and IPAF -/- from Professor Kate Fitzgerald (via Professor Clare Bryant, Cambridge University), University of Massachusetts Medical School and caspase 11 -/- from Professor Dixit, Genentech, USA.

### *In vivo* models

CS or LPS exposure protocols have been described previously [8]. Briefly, mice were exposed to CS (University of Kentucky Research Cigarettes [3R4F] without the filters, 1 hour, twice a day for 3 days) or LPS (Escherichia coli serotype 0111:B4 from Sigma, UK, aerosol of 1 mg/ml for 30 minutes) and the lungs lavaged 24 or 6 hours later, respectively. BALF IL-1α, IL-1β, IL-18, KC, neutrophil and in some cases ATP levels (no increase detected in the LPS model) were assessed as previously described [8]. Tissue caspase 1 activity was assessed in the cytosol fraction of lung homogenates using a specific assay [8].

#### Data analysis

Data are expressed as mean ± S.E.M. of n observations. Statistical significance was determined using either Student's t-test or one-way ANOVA followed by an appropriate post-hoc test, using GraphPad Prism 5 software. A P value <0.05 was taken as significant and all treatments were compared with the appropriate control group.

## Results

### Role of the inflammasome proteins

**Figure 1:**
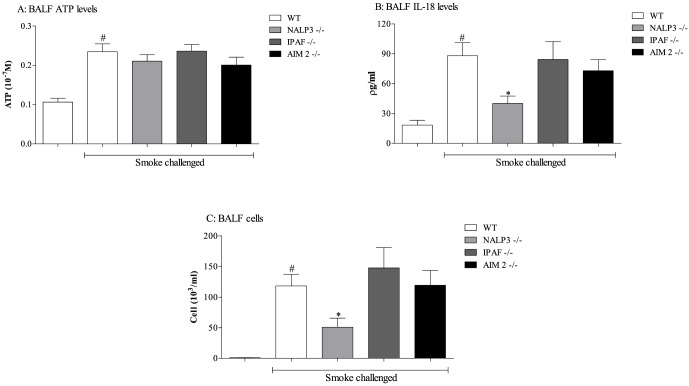
Role of the inflammasome proteins in the CS-driven model. NALP3, IPAF or AIM2 ^-/-^ mice were exposed to CS or room air (control) twice daily for 3 consecutive days alongside wild-type controls. BALF was collected 24 hours after the last exposure for measurement of ATP (A), IL-18 (B) and neutrophil (C) levels. Data are represented as mean ± S.E.M. for n = 8 animals in each group. Statistical significance was determined using Mann-Whitney U test. #  = P<0.05, denoting a significant difference between the smoke exposed and air exposed wild-type groups; *  = P<0.05, denoting a significant difference between the smoke exposed knock-outs and wild-types (one-way ANOVA).

**Figure 2:**
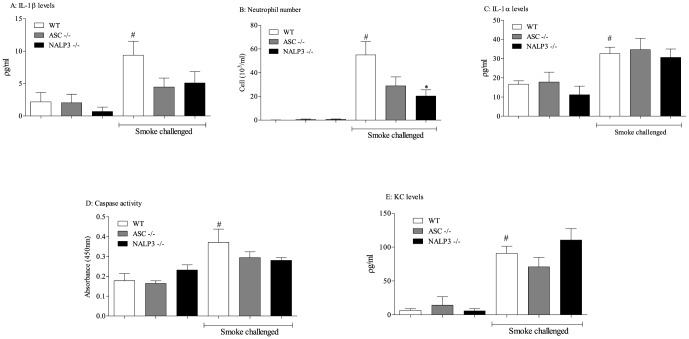
Role of the NALP3 inflammasome in CS-driven model. NALP3 or ASC ^-/-^ mice were exposed to CS or room air (control) twice daily for 3 consecutive days alongside wild-type controls. BALF and lung tissue was collected 24 hours after the last exposure for measurement of IL-1β (A), neutrophil (B), IL-1α (C), caspase 1 activity (D) and KC (E) levels. Data are represented as mean ± S.E.M. for n = 8 animals in each group. Statistical significance was determined using Mann-Whitney U test. #  = P<0.05, denoting a significant difference between the smoke exposed and air exposed wild-type groups; *  = P<0.05, denoting a significant difference between the smoke exposed knock-outs and wild-types (one-way ANOVA).

Exposing wild type mice to 3 days of CS (giving an average total particulate matter of 600–700 mg/M^3^) led to significantly increased levels of ATP, IL-18 and neutrophilia in the BALF (Figure 1). Mice missing functional NALP3 had significantly reduced levels of IL-18 in the BALF and around 50% reduction in BAL neutrophilia, whereas mice missing functional IPAF or AIM 2 were not protected from the CS challenge (Figure 1B and C). As expected ATP levels were not altered in these GM mice compared to wild type mice (Figure 1A). To examine the role of NALP3 further, we repeated that assessment and included mice missing functional ASC, an adaptor molecule that is thought to be required for NALP3 inflammasome activity [35]. Mice missing functional NALP3 and ASC had reduced IL-1β levels in the BALF and again 50% reduction in neutrophilia (Figure 2A and B). Interestingly, the levels of IL-1α were not altered and there was only a small reduction in lung tissue caspase 1 activity. This suggests that IL-1α is not downstream of NALP3/ASC in this system and not all of the tissue caspase 1 activity is dependent on this inflammasome (Figure 2C and D). The CS-induced KC levels were not altered in these GM mice (Figure 2E).

In contrast to the CS driven model, mice missing functional NALP3, ASC, IPAF or AIM 2 responded normally to the LPS challenge. The numbers of neutrophilia in the BALF or lung tissue were not significantly different from the wild type control mice suggesting that these proteins are not required for airway neutrophilic inflammation *per se* (Figure S1).

### Role of caspase 1 and 11

**Figure 3:**
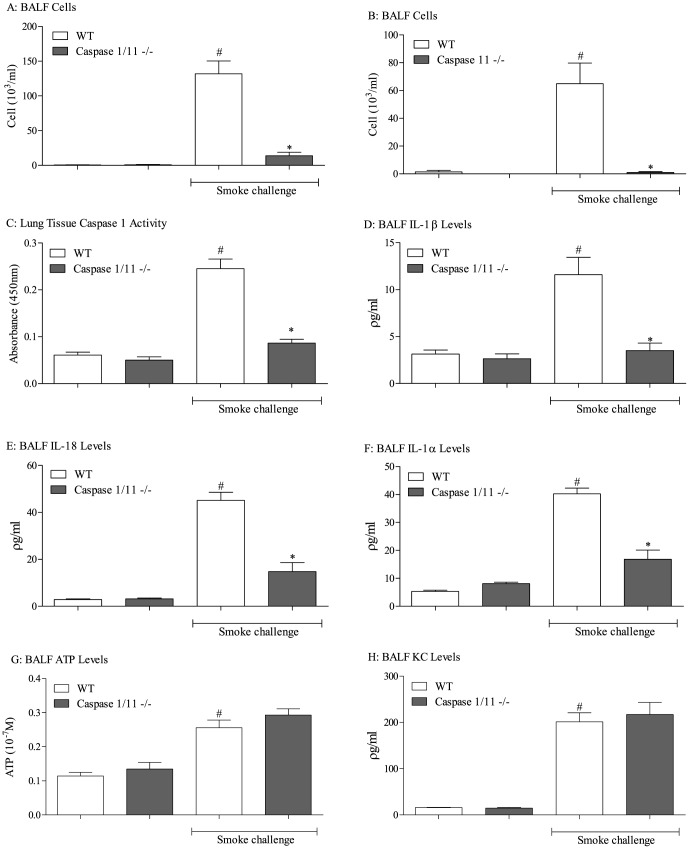
Role of the caspase 1 and 11 in CS-driven model. Caspase 1/11 or caspase 11 ^-/-^ mice were exposed to CS or room air (control) twice daily for 3 consecutive days alongside wild-type controls. BALF and lung tissue was collected 24 hours after the last exposure for measurement of neutrophilia (A – caspase 1/11 -/-, B – caspase 11-/-). Figures C to H depicts the levels of caspase 1 activity (C), IL-1β (D), IL-18 (E), IL-1α (F), ATP (G) and KC (H). Data are represented as mean ± S.E.M. for n = 8 animals in each group. Statistical significance was determined using Mann-Whitney U test. #  = P<0.05, denoting a significant difference between the smoke exposed and air exposed wild-type groups; *  = P<0.05, denoting a significant difference between the smoke exposed knock-outs and wild-types.

The data obtained above suggested that not all lung tissue caspase 1 activity is associated with NALP3/ASC in this model system. To investigate this further we used mice missing functional caspase 1/11 or caspase 11. Both GM mouse lines were completely protected from CS challenge as BALF neutrophilia in challenge groups were not different to that of the unchallenged controls (Figure 3A and B). The caspase KOs had reduced of caspase 1 activity and IL-1β/IL-18 after CS challenge. Interestingly, the levels of IL-1α were also reduced in these mice suggesting that this cytokine was down stream of caspase (Figure 3F). Levels of ATP and KC were not different from wild type controls (Figure 3 G and H). Mice missing functional caspase 1/11 or caspase 11 had equivalent airway inflammation after LPS challenge compared to the wild type control mice suggesting these proteins are not central to airway neutrophilia *per se* (Figure S2).

### Role of IL-1β, IL-1α and IL-18

**Figure 4:**
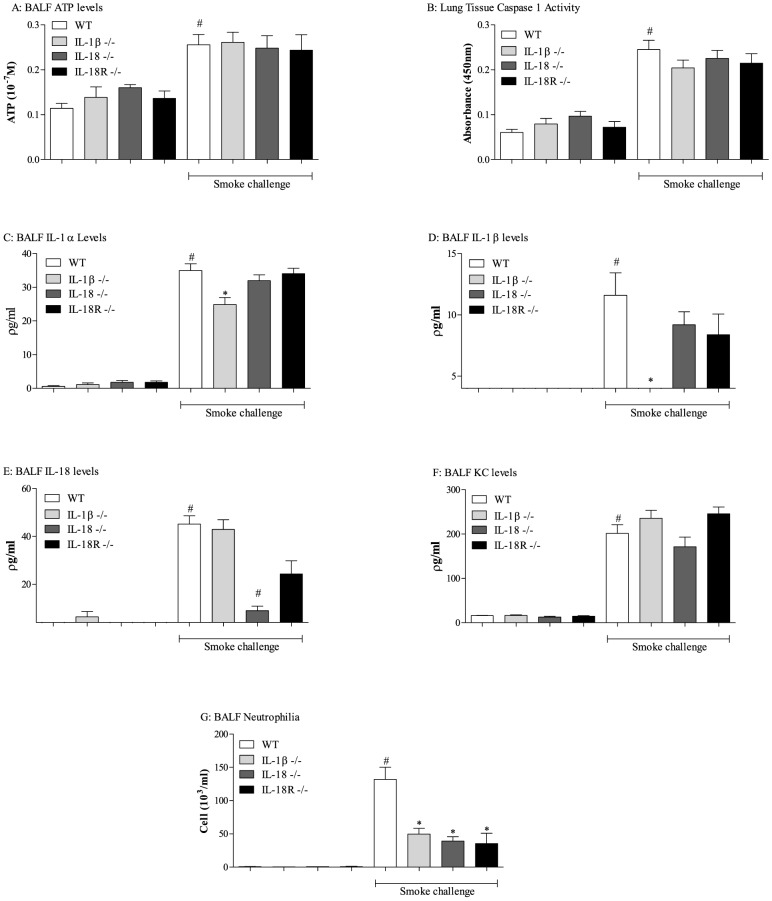
Role of the IL-1 family cytokines in CS-driven model. IL-1β, IL-18 or IL-18R ^-/-^ mice were exposed to CS or room air (control) twice daily for 3 consecutive days alongside wild-type controls. BALF and lung tissue was collected 24 hours after the last exposure for measurement of ATP (A), caspase 1 activity (B), IL-1α (C), IL-1β (D), IL-18 (E), KC (F) and neutrophil (G) levels. Data are represented as mean ± S.E.M. for n = 8 animals in each group. Statistical significance was determined using Mann-Whitney U test. #  = P<0.05, denoting a significant difference between the smoke exposed and air exposed wild-type groups; *  = P<0.05, denoting a significant difference between the smoke exposed knock-outs and wild-types (one-way ANOVA).

The data above suggests that the IL-1 family of cytokines play a key role in airway neutrophilia after CS challenge. To investigate this further we used IL-1β, IL-1α, IL-18 and IL-18R KO mice. CS challenge caused a significant increase in ATP, caspase 1 activity, IL-1β, IL-1α, IL-18, KC and neutrophilia in the control wild type mice (Figure 4). IL-1β and IL-18 levels were significantly reduced in the corresponding KO lines (Figure 4D and E). IL-1α levels were slightly reduced in the IL-1β KO mice. There was a round a 50% reduction in neutrophilia in mice missing IL-1β, IL-18 and IL-18R but levels of ATP, caspase 1 activity and KC were not altered (Figure 4G). Mice missing functional IL-1β or IL-18 did not have altered airway neutrophilia after LPS challenge (Figure S3).

After CS challenge the IL-1α KO mice had reduced IL-1α, IL-1β and IL-18 in the BALF and surprisingly completely attenuated neutrophilia (Figure S4). Levels of ATP, caspase 1 activity and KC were not altered (Figure 4A, B and F). Unlike the other GM lines we used in this study, we found that mice missing functional IL-1α had significantly less neutrophil numbers in the lung tissue under basal conditions (data from 2 separate experiments: study one 1: Wild type – 9940±1392, IL-1α KO – 4804±359 neutrophils/mg of lung tissue; study one 2: Wild type – 5967±804, IL-1α KO – 3908±213 neutrophils/mg of lung tissue). We suggest that this phenotypic change observed in this GM line is likely to complicate data interpretation in the disease models. It would seem likely that some of the BALF neutrophilia and perhaps cytokines could be influenced by tissue levels. Indeed, the IL-1α KO mice had reduced neutrophilia after LPS challenge (data not shown).

### Role of IL-1α, IL-1β and IL-18 on neutrophil transmigration

**Figure 5:**
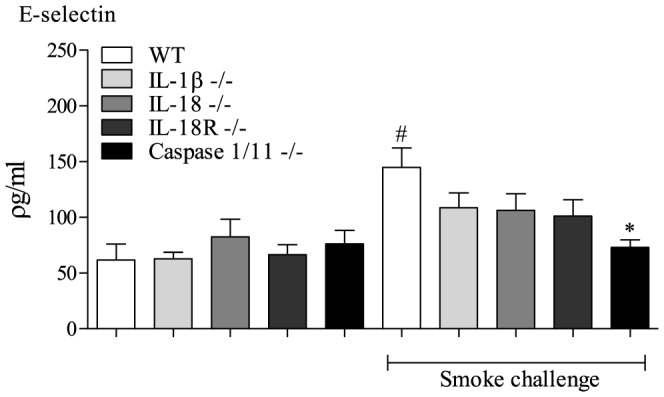
Role of the IL-1β and IL-18 in CS-driven model. IL-1β, IL-18, IL-18R or caspase 1/11 ^-/-^ mice were exposed to CS or room air (control) twice daily for 3 consecutive days alongside wild-type controls. Lung tissue was collected 24 hours after the last exposure for measurement of E-selectin levels. Data are represented as mean ± S.E.M. for n = 8 animals in each group. Statistical significance was determined using Mann-Whitney U test. #  = P<0.05, denoting a significant difference between the smoke exposed and air exposed wild-type groups; *  = P<0.05, denoting a significant difference between the smoke exposed knock-outs and wild-types (one-way ANOVA).

Lastly we asked how these cytokines could be involved in the BALF neutrophilia we observe after CS challenge. These cytokines are not thought to be direct chemoattractants but involved in the expression of transmigration proteins such as E-selectin [36]–[38]. CS caused a significant increase in E-selectin protein levels in CS exposed lung tissue (Figure 5). These levels were partially reduced in mice missing IL-1β, IL-18 or IL-18 (the GM lines that had 50% reduction in CS-induced neutrophilia) and completely attenuated levels in the caspase 1/11 KO mice (the GM line that had complete attenuation of neutrophilia and a reduction in IL-1α, IL-1β or IL-18) (Figure 5). Together this data suggest a causative link between levels of the IL-1 family cytokines, expression of transmigration proteins and the level of neutrophilia.

## Discussion

COPD is a chronic inflammatory airway disease with no effective therapy [2], [3]. Current dogma suggests that in a majority of patients CS exposure is the causative agent which initiates the inflammatory response leading to disease pathogenesis. Therefore, reducing the inflammation should lead to a decline in disease progression and possibly a reduction in symptoms. Currently significant research focus and pharmaceutical company direction is geared towards the discovery of effective anti-inflammatory therapeutic targets given the minimal efficacy of existing treatments [4]. One approach to elucidate novel targets is to examine the mechanism by which CS exposure causes airway inflammation. Previously we, and others, have shown that ATP activation of the P2X_7_ receptor plays a key role in acute and chronic murine models of CS-induced airway inflammation/emphysema [8], [9]. The aim of this study was to investigate the post P2X_7_ receptor signalling pathway.

**Figure 6:**
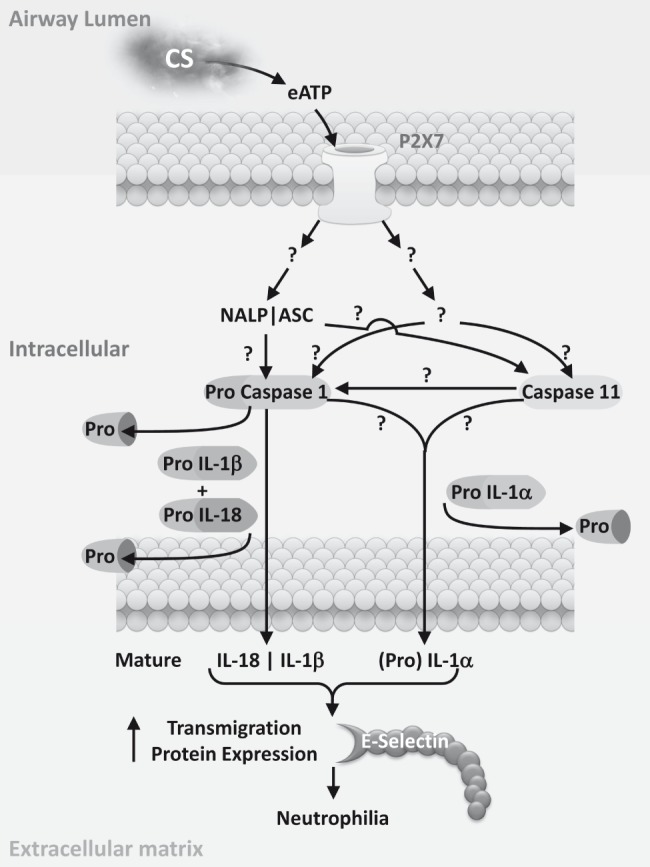
Schematic representation of the signalling cascade following CS-induced activation of the P2X_7_ channel in the lung (Drawn by Dr E Dubuis). We hypothesise that CS exposure leads to the release of extracellular ATP which activates P2X_7_ receptor, and this in turn triggers a signalling cascade involving the assembly of the NALP3/ASC inflammasome and recruitment of (pro)-caspase 1. Pro-Caspase 1 either auto-processes itself to the mature, active form or is cleaved by caspase 11. This functional inflammasome is essential for the release of mature, active IL-1β/IL-18, but not IL-1α which stimulates some of the production of proteins involved in the transmigration of neutrophils, like E-selectin. Another component of the P2X_7_ receptor signalling cascade triggers the caspase 1 and/or caspase 11 dependent release of IL-1α. This cytokine also induces the production of proteins involved in the recruitment of a portion of the neutrophils observed in this model system. Interrupting steps involved in both signalling pathways i.e. at the P2X_7_ receptor or via caspase 1/11 leads to a complete block of transmigration protein production and neutrophilia.

In this study we found that CS induced ATP activation of the P2X_7_ receptor appears to trigger the formation of the NALP3/ASC inflammasome and recruitment of caspase 1. This leads to maturation of pro-IL-1β/IL-18 and accounts for some of the CS induced airway neutrophilia. Interestingly, the NALP3 inflammasome was not essential for IL-1α release; this cytokine, however, did appear to be under the control of caspase 1/11. Further, CS-induced neutrophilia was completely attenuated in mice missing functional caspase 1/11 or just caspase 11. This suggested that the neutrophilia observed in this model is driven via a combination of signals from IL-1β/IL-18 and IL-1α. Indeed, mice missing IL-1β or IL-18 had partially reduced levels of CS-induced neutrophilia. Furthermore, the reason why IL-1β/IL-18 and IL-1α were required for neutrophilia seemed to be associated with production of proteins involved in cell transmigration into the airway lumen i.e. E-selectin. A schematic diagram illustrating the proposed signalling cascade following CS-induced activation of the P2X_7_ channel in the lung is shown in Figure 6.

Initial studies were aimed at exploring the role of the various inflammasome proteins in the production of the three IL-1 cytokines [27]–[29]. Data showed that NALP3, and its essential co-factor ASC, were required for the release of IL-1β and IL-18 into the BALF. This was associated with approximately 50% reduction in BALF neutrophilia, suggesting that these two cytokines are responsible for approximately half of the cellular recruitment. Interestingly, the levels of IL-1α were not altered in these GM mice and caspase 1 activity was only reduced by around 50%. This suggested that IL-1α and a portion of the caspase 1 activity in this model system are under the control of a different signalling mechanism. This difference in control of IL-1α and IL-1β in smoke systems has been reported by others [20]. To explore this further we employed mice missing functional caspase 1 or caspase 11. Recently it has been established that due to the way they were originally engineered, the caspase 1 KO mice are also deficient in caspase 11 [32]. Whilst caspase 11 is not thought to process pro-IL-1β directly, it has been reported to cleave pro-caspase 1 into the mature, active form and it has also been linked to the release of IL-1α [30]–[34]. In these studies, the levels of neutrophilia in mice missing caspase 1/11 or just caspase 11 after CS challenge were similar to that of the air challenged control. This suggested that they play an essential role in the CS-induced ATP-P2X_7_ signalling. Indeed, further investigation showed that the attenuation of neutrophilia was associated with a reduction in lung tissue caspase 1 activity, IL-1β/IL-18 and importantly IL-1α. This is further evidence that in this model IL-1β/IL-18 release is via NALP3/ASC activation of caspase 1, whereas IL-1α release is via an as yet under known signal between the P2X_7_ receptor and caspase 11. Furthermore it suggests that blockade of IL-1β/IL-18 and IL-1α is required for complete suppression of CS-induced neutrophilia. Interestingly, other groups have suggested that mice missing caspase 1 (it is not clear if these mice are the specific caspase 1 or dual KOs) do not have reduced neutrophilia [18], [20] and others have reported that a caspase 1 inhibitor does reduce CS induced airway inflammation [39]. It is not clear why this discrepancy exists.

To investigate this further, we performed CS challenge in mice missing IL-1β, IL-18, IL-18R and IL-1α. The data showed that the respective cytokines were missing in the appropriate gene deleted mice and that the IL-1β KO mice had a small reduction in IL-1α too. The IL-1β, IL-18 or IL-18R KOs had around 50% reduction in BALF neutrophilia, which was very reminiscent of the profile seen in the NALP3/ASC KO mice (that had reduction in both IL-1β and IL-18). This suggests that perhaps IL-1β and IL-18 are essential for this portion of the neutrophilia and that a reduction in either would have a functional effect. Indeed, similarly others have published similar levels of inhibition when they targeted either IL-1β or IL-18 [10], [16], [20], [39]–[43]. Furthermore, when either IL-1β and IL-18 is over expressed it leads to emphysema/COPD [44]–[46]. Further evidence that IL-1β and IL-18 are essential comes from the data demonstrating that CS challenge increased total E-selectin levels and mice missing IL-1β, IL-18 or IL-18R exhibited an inhibition profile similar to the neutrophilia. This data also suggests that, as hypothesised, the role of these cytokines in this model system is in the upregulation of proteins involved in neutrophil transmigration into the airway lumen [36]–[38]. Indeed, further evidence for this comes from the data from the mice missing caspase 1/11, which had reduced levels of IL-1β/IL-18 and IL-1α. The levels of E-selectin, like the neutrophilia, in these mice were completely attenuated. This again would suggest that IL-1α plays a dominant role in CS-induced neutrophilia. To examine this we profiled IL-1α KO mice and found a dramatic reduction in neutrophilia which was associated with attenuation of the IL-1α signal, and some reduction in IL-1β and IL-18. This role of IL-1α has been suggested before [16], [20], however we were intrigued by the magnitude of the inhibition as based on the other data obtained we would have expected a 50% reduction in these mice. A reason for this larger effect on neutrophilia could be due the phenotype of the mice under basal conditions. In two separate experiments we noted that the mice missing IL-1α had significantly less neutrophils in the lung tissue. We speculate that that some of the inhibition observed in the CS model, and the LPS model, could be due to the fact there are less neutrophils in the nearby tissue to migrate into the airway lumen and as such we suggest this data is interpreted with caution.

In these studies we could detect an increase in BALF ATP and KC levels after CS challenge but the mechanisms involved are not known [7], [8]. However, what is clear from these studies is that both these mediators are not controlled by the signalling downstream of the P2X_7_ receptor and are independent of neutrophilia. Furthermore, in the LPS studies the numbers of neutrophils were not altered in any of the GM lines employed suggesting that the reduction in neutrophilia seen in the CS model is not a generic effect on airway neutrophilia.

In conclusion, this data suggests that airway neutrophilia induced by exposure to CS is via the maturation and release of IL-1β/IL-18 and IL-1α. The release of IL-1β/IL-18 is dependent on the NALP3/ASC inflammasome and caspase 1/11 activity, whereas IL-1α appears to involve caspase 1/11 independently of IPAF, AIM2 and NALP3 (for schematic see Figure 6). Whilst these observations were made in an acute CS-driven model, which predominantly involves neutrophilia and not macrophages (believed to be central to the pathogenesis of COPD, [47]–[49], we suggest that these data represents an important step in understanding the mechanism by which CS exposure leads to airway inflammation and starts to highlight possible new therapeutic avenues for the treatment of COPD.

## Supporting Information

Figure S1 Role of the inflammasome proteins in LPS-driven model. ASC, NALP3, IPAF or AIM2 -/- mice were exposed to aerosolised LPS (1 mg/ml) or vehicle (saline) for 30 minutes alongside wild-type controls. BALF and lung tissue were collected 6 hours after the exposure for measurement of neutrophilia (BALF - A; tissue - B). Data are represented as mean ± S.E.M. for n = 8 animals in each group. Statistical significance was determined using Mann-Whitney U test. #  = P<0.05, denoting a significant difference between the smoke exposed and air exposed wild-type groups; *  = P<0.05, denoting a significant difference between the LPS exposed knock-outs and wild-types (one-way ANOVA).
(EPS)

Figure S2 Role of caspase 1 and 11 in LPS-driven model. Caspase 1/11 or caspase 11 -/- mice were exposed to aerosolised LPS (1 mg/ml) or vehicle (saline) for 30 minutes alongside wild-type controls. BALF and lung tissue were collected 6 hours after the exposure for measurement of neutrophilia (BALF – A and C; tissue – B and D). Data are represented as mean ± S.E.M. for n = 8 animals in each group. Statistical significance was determined using Mann-Whitney U test. #  = P<0.05, denoting a significant difference between the smoke exposed and air exposed wild-type groups; *  = P<0.05, denoting a significant difference between the LPS exposed knock-outs and wild-types.
(EPS)

Figure S3 Role of IL-1 family cytokines in LPS-driven model. IL-1β or IL-18 mice were exposed to aerosolised LPS (1 mg/ml) or vehicle (saline) for 30 minutes alongside wild-type controls. BALF and lung tissue were collected 6 hours after the exposure for measurement of neutrophilia (BALF – A; tissue – B). Data are represented as mean ± S.E.M. for n = 8 animals in each group. Statistical significance was determined using Mann-Whitney U test. #  = P<0.05, denoting a significant difference between the smoke exposed and air exposed wild-type groups; *  = P<0.05, denoting a significant difference between the LPS exposed knock-outs and wild-types (one-way ANOVA).
(EPS)

Figure S4 Role of the IL-1α in CS-driven model. IL-1α ^-/-^ mice were exposed to CS or room air (control) twice daily for 3 consecutive days alongside wild-type controls. BALF and lung tissue was collected 24 hours after the last exposure for measurement of ATP (A), caspase 1 activity (B), IL-1α (C), IL-1β (D), IL-18 (E), KC (F) and neutrophil (G) levels. Data are represented as mean ± S.E.M. for n = 8 animals in each group. Statistical significance was determined using Mann-Whitney U test. #  = P<0.05, denoting a significant difference between the smoke exposed and air exposed wild-type groups; *  = P<0.05, denoting a significant difference between the smoke exposed knock-outs and wild-types.
(EPS)
